# Exploring the Potentialities of Photoinduced Glycation to Steer Protein Functionalities: The Study Case of Freeze-Dried Egg White Proteins/Carbohydrates Mixtures

**DOI:** 10.3390/foods10010026

**Published:** 2020-12-24

**Authors:** Lara Manzocco, Stella Plazzotta, Sonia Calligaris

**Affiliations:** Department of Agricultural, Food, Environmental and Animal Sciences, University of Udine, Via Sondrio 2/A, 33100 Udine, Italy; stella.plazzotta@uniud.it (S.P.); sonia.calligaris@uniud.it (S.C.)

**Keywords:** ultraviolet, UV-C, Maillard reaction, proteins, viscosity, foams

## Abstract

The capacity of UV-C light to induce glycation and modify functional properties of systems containing freeze-dried egg white proteins and carbohydrates with increasing molecular weight (i.e., glucose, maltose, trehalose and maltodextrin) was studied. Color changes induced by light exposure were taken as typical indicators of glycation. Samples were then analyzed for selected physical (critical concentration, particle size and viscosity), chemical (ovalbumin content) and technofunctional properties (gelling temperature and foaming capacity). The presence of sugars during exposure to UV-C light promoted intense browning and decreased ovalbumin content by circa 30%. Concomitantly, up to a 3-fold increase in critical concentration of the aqueous suspensions of the irradiated protein-carbohydrate powders and changes in particle size were detected. These modifications were consistent with the development of non-enzymatic browning reactions upon UV-C light irradiation. Photoinduced glycation was associated to a decrease in viscosity, a tendency to form gel at temperature lower by up to 8 °C and a better capacity of foam stabilization. The intensity of these changes seems to be affected by the nature of the carbohydrates reacting with proteins, with longer carbohydrates able to produce systems with higher foam stability capacity.

## 1. Introduction

UV-C treatments have been proposed as a viable non thermal alternative in liquid and solid foods for eliminating or reducing the occurrence of undesirable microorganisms. Much less is known about the interaction of UV light with food components. Most of the literature refers to proteins, which are known to easily undergo chemical modification following exposure to UV-C light. Their photoreactivity is due to the abundance of chromophores (e.g., tryptophan, tyrosine, phenylalanine, cysteine, flavins and heme), able to rapidly react with other excited state species [1]. The energy of the radiation absorbed by the chromophores is transferred between the initial sites of oxidation and the part of the protein that controls its structure and function. This transfer is primarily directed to sulphur-containing amino acids through hydrogen bonding networks and protein backbone [2,3]. As a result, side-chain oxidation, backbone fragmentation and/or formation of cross-links and aggregates occur, leading to a modification in both protein structure and properties [4]. For instance, UV irradiation was suggested to photocross-link proteins, making them more resistant to biodegradation or modifying their rheological and gelling properties [5,6,7,8]. In addition, UV-C treatments demonstrated the potential for improving antioxidant activity in β-lactoglobulin and, in the case of egg white, exerted a positive impact on foam ability, foam stability, emulsifying activity and immunoreactivity [9,10,11].

Carbohydrates are generally poor absorbers of UV radiation. However, their photocross-link and/or depolymerization might occur, triggered by the presence of chromophores such as proteins and non-enzymatic browning products [12,13]. These are typical contaminants deriving from treatments performed during carbohydrate production and hydrolysis. Similar to proteins, also carbohydrates might undergo structural and property changes upon photoreaction. In the case of starch, improved mechanic performance, higher viscosity and delayed swelling, solubility and retrogradation were reported [14,15,16].

The concomitant presence of proteins and carbohydrates is likely to critically affect the photoreactivity of the system. From one side, the presence of large polysaccharides would reduce protein photoreactivity by hindering photoinduced structure modifications of the protein fraction [3]. On the other hand, light treatments of protein rich systems containing sugars have been shown to promote Maillard reaction and cross-linking [6,10,17,18,19].

The development of the Maillard reaction has been also proposed as a method to modify protein functionalities [20]. Kchaou et al. [21] demonstrated that the development of Maillard reaction improved the mechanical properties of gelatin films prepared by incorporation of glucose. Glycation of chicken and whey proteins with sugars and dextran, respectively, modified proteins and their antigen-binding ability [22,23]. Ovalbumin emulsifying capacity was also improved by glycation via the Mallard reaction with monosaccharides and disaccharides [24,25]. It is noteworthy that the above mentioned results were all achieved by inducing Maillard reaction by heating. Nevertheless, similar results could be potentially achieved using UV-C light instead of thermal treatments. Despite the potential of light processing in steering protein/carbohydrate glycation, surprisingly no information on this topic is available and no indication is provided about the effects of photoinduced glycation on protein functionalities.

Based on these considerations, the aim of this work was to investigate the capacity of UV-C light to induce glycation and modify functional properties of systems containing proteins and carbohydrates. To this aim, model systems containing egg white proteins and carbohydrates with increasing molecular weight (i.e., glucose, maltose, trehalose and maltodextrin) were considered. Color changes induced by light exposure were taken as typical indicators of glycation. Samples were then analyzed for selected physical (critical concentration, particle size and viscosity), chemical (ovalbumin content) and technofunctional (gelling temperature, foaming capacity) properties.

## 2. Materials and Methods

### 2.1. Materials

Egg white (egg whites from chicken, Sigma-Aldrich, Milan, Italy); glucose (J.T. Baker, Milan, Italy); maltose (Sigma-Aldrich, Milan, Italy); trehalose (Britishsugar, Peterborough, UK); maltodextrin 18.5 DE (Dry MD 18.5 DE, Cerestar Italia S.p.a., Milan, Italy); KH_2_PO_4_ (Sigma Aldrich, Milan, Italy); K_2_HPO_4_ (J.T. Baker, Milan, Italy); bovine serum albumin (67 kDa; Sigma, St. Louis, MO, USA); β-lactoglobulin (18 kDa; Sigma, St. Louis, MO, USA) and albumin (44.29 kDa; Sigma, St. Louis, MO, USA).

### 2.2. Sample Preparation

Aqueous solutions containing 40% (*w*/*w*) of dry samples were prepared by stirring at room temperature for 30 min. The samples considered were: egg white, glucose, maltose, trehalose and maltodextrin and a 1:3 (*w*/*w*) mixture of egg white and one of the sugars (egg white–glucose; egg white–maltose and egg white–trehalose). The solutions were frozen at −80 °C (Forma 900 Series, Thermo Fisher Scientific, Outside, Waltham, MA, USA), freeze-dried (Edwards Alto Vuoto S.p.a., Trezzano sul Naviglio, Milan, Italy), immediately introduced in desiccators containing phosphorus pentoxide (Sigma-Aldrich, Milan, Italy) and maintained at 25 °C in the dark until use.

### 2.3. UV-C Light Treatments

Aliquots of 0.18 g of freeze dried sample were introduced into 5 × 10 cm plastic pouches (polycoupled Combiflex PA/PE 090, 20/70, Savonitti, Codroipo, Italy) and hermetically sealed (Orved, VM-16, Musile di Piave, Italy). Samples were immediately exposed for 4, 24 and 72 h to the light emitted by two UV-C lamps (15 W, G15T8, Philips, Holland) in a thermostated cell (POL-EKO-APARATURA SP.J., ul. Kokoszycka 172C, 44-300 Wodzlslaw SI. POLSKA) at 20 °C. The lamps were allowed to stabilize by turning them on at least 30 min before use. Samples were positioned at the same distance between the two lamps, so that 20 W m^−2^ UV-C light irradiance was achieved. No temperature changes were observed as a consequence of lighting in all samples. Analogous samples not submitted to UV-C light were also prepared as control.

### 2.4. Irradiance

Irradiance was measured using a portable luminometer (HD-2102.2 Delta Ohm, Padova, Italy) equipped with a UV-C light probe (LP471 UVC, Padova, Italy).

### 2.5. Color

Powders were placed in Petri dishes (6–7 cm diameter) and analyzed for color using a tristimulus colorimeter (Chromameter, CR-400/410 Ver. 1.03, Minolta Co., Ltd., Osaka, Japan) equipped with a CR-300 measuring head. The instrument was standardized against a white tile before measurements. Color was expressed in L*, a* and b* Hunter scale parameters. The overall color differences (ΔE*) were calculated using Equation (1), where the suffixes “*o*” denotes untreated samples.

(1) $$\Delta E^{*}=\sqrt{{\left(L^{*}-L_{o}^{*}\right)}^{2}+{\left(a^{*}-a_{o}^{*}\right)}^{2}+{\left(b^{*}-b_{o}^{*}\right)}^{2}}\;$$

### 2.6. Solubility

Powders were suspended in Milli-Q water at a 5% (*w*/*v*) concentration, treated with ultrasounds (Ultrasonic Cleaner, USC 900 D, VWR international, Milan, Italy) for 30 min at 25 °C to facilitate the dispersion of the powder and filtered once through (250 μm porosity, Millipore, Burlington, VT, USA). The percentage of sample mass flowing downstream from the filter was then measured by determining the total solid content of the filtered sample. To this aim, filtered samples were accurately weighted in previously dried dishes and dried to constant weight.

### 2.7. HPLC Gel-Permeation Analysis

Samples were filtered on 0.20 μm porosity filters (Econofilters, Agilent Technologies, Cernusco sul Naviglio, Italy) and analyzed using the HPLC system Varian Prostar (model 230, Varian Associates Ltd., Walnut Creek, CA, USA), photodiode array detector (220 nm), Varian Prostar Software, “Jerry Varian” method. A precolumn, protecting 3 mm ID column KJ0-4282, Security GuardTM (Phenomenex, Torrance, CA, USA), equipped with two columns, BioSep-SEC-S 3000 and BioSep-SEC-S 2000, 30 cm length, 7.80 mm internal diameter, 5 μm granulometry and 125 Å porosity, with a separation range between 5 and 150 kDa, (Phenomenex, Torrance, CA, USA) were used. Injection volume was 20 μL and the mobile phase, delivered at a flow rate of 0.6 mL min^−1^, was 0.1 M phosphate buffer pH 7.0 in isocratic condition. Detection wavelength was 220 nm. Bovine serum albumin (67 kDa), β-lactoglobulin (18 kDa) and albumin (44.29 kDa) were used as calibration standards. A linear relation (R^2^ = 0.99) was found between retention time and molecular weight of standard proteins, expressed in logarithmic values. Peaks integration was performed by CHROM-CARD for Windows software (1.19 version).

### 2.8. Functional Properties

#### 2.8.1. Critical Concentration

Viscosity was evaluated by using a capillary viscometer (size 50, Cannon-Fenske Routine Viscometers, UK) at 25.0 ± 0.1 °C. Time to flow, expressed in s, was related to egg white concentration *C* (g/L) according to the equation of a straight line (R^2^ = 0.99). Relative viscosity (*η_rel_*) was calculated as follows:

(2) $$\eta_{rel}={\frac{t}{t}}_{s}$$

where *t* is the time to flow of the suspension and *t_s_* is the time to flow of the solvent. Specific viscosity (*η_sp_* = *η_rel_* − 1) and reduced viscosity $\left(\frac{\eta_{sp}}{C}\right)$, expressed as L/g, were then computed.

The critical concentration C*, expressed as g/L, was computed as the reciprocal value of reduced viscosity.

#### 2.8.2. Particle Size

Light scattering measures were made using a particle sizer NICOMP™ 380 ZLS (PSS NICOMP Particle Sizing System, Santa Barbara, CA, USA). Samples were diluted 1:1000 (*w*/*v*) with Milli-Q water and treated with ultrasound (Ultrasonic Cleaner, USC 900 D, VWR international, Milan, Italy) for 30 min at 25 °C to facilitate dispersion. The angle of observation was 90°. The refractive index of the solution was set at 1.333 and the viscosity was approximated to that of pure water at 25 °C. The hydrodynamic radius refers to the corresponding volume distribution calculated by NICOMP Distribution Analysis. Results were reported as the peak value ± the symmetrical dispersion from the 10 to the 90 percentile of the distribution.

#### 2.8.3. Apparent Viscosity

Apparent viscosity at 20 °C was measured by a RS6000 Rheometer (ThermoScientific Rheo Stress, Haake, Germany) equipped with a Peltier system for temperature control. Parallel plate geometry of 40 mm diameter was used with a gap of 1 mm. The measurements were performed at shear rate from 0.01 to 200 s^−1^. Apparent viscosity was calculated at a shear rate equal to 0.1 s^−1^.

#### 2.8.4. Gelling Temperature

Gelling temperature was determined by a rotational rheometer (Stresstech Rheometer, Reologia Instruments AB, Lund, Sweden) equipped with 40 mm diameter cone-plate geometry. As reported by Raikos et al. [26], a temperature sweep was conducted from 60 to 75 °C at a heating rate of 1.0 °C min^−1^ using a frequency of 1 Hz and a strain of 0.008. These conditions were chosen within the linear viscoelastic region of the samples. The outer edges of the plates were covered with a thin layer of mineral oil (d = 0.84 g mL^−1^, Sigma Aldrich, Milan, Italy) to minimize water loss during measurements.

#### 2.8.5. Foam Ability and Stability

Foams were processed by whipping 5 mL of egg white for 3 min at 20 °C in a 50 mL cylinder by a homogenizer (Polytron, PT 3000, Cinematica, Littau, Switzerland) operating at 9500 rpm. The volumes of the foam and of the drained liquid were assessed just after whipping and after holding for 30 min at 20 °C. Foam stability was calculated as the percentage ratio between the foam volume at observation time and that detected just after whipping.

### 2.9. Statistical Analysis

All determinations were expressed as the mean ± standard deviation (SD) of at least three repeated measurements from two experiment replicates (*n* = 2). Statistical analysis was performed by using R v. 2.15.0 (The R Foundation for Statistical Computing, Wien, Austria). Bartlett’s test was used to check the homogeneity of variance, a one-way ANOVA was carried out and Tukey test was used to determine statistically significant differences among means (*p* < 0.05).

## 3. Results and Discussion

### 3.1. Browning Development

Visual observation and colorimetric analysis of samples indicated that exposure to UV-C light made sample color turn from white to amber tones (Table 1). Color difference in all samples gradually increased with the time of UV-C light exposure. Lower color changes were observed in systems containing only sugars. This is consistent with the fact that sugars are poor absorbers of UV radiation. In any case, it is expected that they contain chromophores deriving from the production process. These chromophores may act as photosensitizers, inducing a certain level of photoresponse, probably leading to non-enzymatic browning reactions similar to those induced by thermal caramelization. Opposite results were reported for sugar syrups and fruit juices exposed to UV-light [27,28]. In these samples, the decrease in color and 5-hydroxymethylfurfural was detected. These contradictory results indicate that photoinduced reactions in sugar powders follow different mechanism from those observed with sugars solubilized in an aqueous phase. In addition, the effect of the difference in emission spectra and output power of UV-C lamps could also account for different color trends.

Color changes resulted more intense during light exposure of egg white (Table 1). Protein photoreaction has actually been reported to form brown pigments in different conditions. For instance, soy protein, gluten, albumin and caseinate films exposed to UV-C light developed a yellowish coloration, which linearly increased with the light dose [6,17]. A similar effect was also observed in egg white and fresh egg pasta [10,19]. Literature evidences relevant to the effect of UV-C light on aqueous suspensions of protein indicate that pigment formation would result from the concomitant development of a number of different photoreactions. Light would actually induce modification of protein binding forces, affecting the secondary and tertiary structure and easily forming high molecular weight aggregates by radical–radical termination reactions or formation of cross-links [1,4,29]. Intermolecular interactions, unfolding and protein cleavage have also been reported [4,10,30,31,32,33]. Based on these considerations, it can be inferred that photoreactions similar to those occurring in the hydrated protein systems could also develop in freeze dried egg white, giving reason for its color changes (Table 1). The presence of sugars made the browning of egg white more intense. To this regard, Manzocco et al. [10,19] observed that UV-C irradiation of model systems containing amino acids and reducing sugars was associated to the formation of a number of different Maillard reaction products. It is thus not excluded that sugars, although being poorly capable of absorbing ultraviolet light, could take part to egg white photoreactions through pathways other than those experienced by the protein solely.

To study the development of photoinduced reactions, protein content was determined by HPLC analysis, focusing the attention on the effect of UV-C light on ovalbumin, that is the main protein of egg white. The percentage of ovalbumin reduction after 72 h of reaction of egg white and egg white in the presence of sugars is reported in Table 1. The extent of ovalbumin decrease in an egg white sample was in the same magnitude range reported in the literature for egg white diluted solutions [34]. The presence of sugars decreased ovalbumin tendency to undergo photoinduced denaturation. This effect is probably due to the fact that ovalbumin would react with carbohydrates rather than simply undergo unfolding. In addition, ovalbumin unfolding could be further hindered due to grafting with carbohydrate moieties. The acquired results on color changes and decrease in ovalbumin content confirm the hypothesis that exposure to UV-C light can be regarded as a possible approach, alternative to heat treatments, to induce the development of glycation reaction in powders of proteins and carbohydrates.

### 3.2. Functional Properties

In order to get more information about photoreaction products of egg white and sugar, freeze-dried powders were suspended in water at a concentration of 5%. The percentage of sample mass that was solubilized in the aqueous phase under the conditions used in this study is reported in Table 2. In the case of the untreated egg white, circa 70% of the sample mass introduced in water was actually recovered downstream from the filter, indicating that most sample was homogeneously hydrated while only a minor part was held on the filter due to the presence of particles with a higher size than that of the filter pores. By contrast, after UV-C light treatment, only 17% of the egg white sample mass was recovered after filtration. In other words, almost 80% of the sample was represented by large protein aggregates, unable to disperse in water and flow downstream from the filter. It is interesting to note that the presence of sugar during the UV-C light treatment of egg white largely decreased particle retention on the filter, indicating a lower presence of large aggregates.

The attention was thus focused on the filtered suspensions, containing the fraction of photoreaction products, which could be easily suspended in water. In order to obtain specific information about these suspensions, the critical concentration (C*) of filtered samples was determined (Table 3). This parameter represents the quantity of macromolecules that can be accommodated in a given volume of suspension without mutual structure perturbation [34]. The critical concentration of untreated egg white was in the range reported in the literature for globular proteins [35]. Light treatment increased the critical concentration of egg white by two orders of magnitude, indicating that a higher amount of sample mass could be allocated in a given volume of suspension. The structure of egg white photoreaction products would thus favor vicinity and interaction among particles, resulting in a lower excluded volume. This could be consistent with the occurrence of cross-linking/unfolding phenomena, leading to protein interpenetration and overlapping. The hypothesis was supported by the dynamic light scattering results on the hydrodynamic radius of the particles (Table 3). The particle size analysis showed in all cases multimodal distributions with the presence of two main peaks associated to two distinct populations: one at low values of diameters (population 1) and the other at higher size (population 2). These populations can be attributed to the presence of dispersed proteins (population 1) and their aggregates (population 2), respectively. It can be noted in Table 3 an increase in hydrodynamic volume of the two particle families as a consequence of the UV-light treatment. Based on these results, light exposure would promote egg white protein modification with formation of novel compounds having larger size and more compact structure. They also embed chromophore groups, probably deriving from intramolecular or intermolecular reactions (Table 1 and Table 2). The presence of carbohydrates significantly increased the critical concentration of the untreated suspension (Table 3). However, the higher the molecular weight of the carbohydrate, the lower the critical concentration. The simple addition of carbohydrates to protein systems is known to be thermodynamically unfavored as they tend to be excluded from the domain of protein molecules [36]. This is due to the polyelectrolyte nature of carbohydrates that beget repulsive electrostatic double-layer forces with proteins. Following UV-induced glycation, proteins are modified into more compact structures, as also indicated by the lower hydrodynamic volume of protein particles (population I) [37,38]. This effect would be particularly intense for small sugars, such as glucose, which can easily come close to the protein surface to undergo glycosilation. By contrast, this effect would be less intense for larger polysaccharides, such as maltodextrin, due to steric hindrance. In these conditions, protein aggregates with higher hydrodynamic volume (population II) would be formed. The formation of aggregates could be attributed not only to protein cross-linking and formation of more compact structures, as already observed for egg whites solely, but also to glycation and formation of protein-carbohydrate aggregates. Carbohydrates seem to exert different effects depending on their nature. In the presence of small sugars (i.e., glucose, threalose and maltose), the photoinduced increase in the critical concentration was associated with an increase of hydrodynamic radius of population I and a decrease of the hydrodynamic volume of population II. It can be inferred that photoinduced reactions of proteins may form interpenetrated compounds, which also include sugar moieties. The latter would have a lower capability of forming large aggregates. By contrast, when maltodextrin was present, very small particles of both protein and protein aggregates were present. These results confirm that egg white sensitivity to UV-C light is definitely affected by the presence of carbohydrates, which can steer reaction pathway leading to the formation of photoreaction products with considerably different chemical structure.

**Figure 1:**
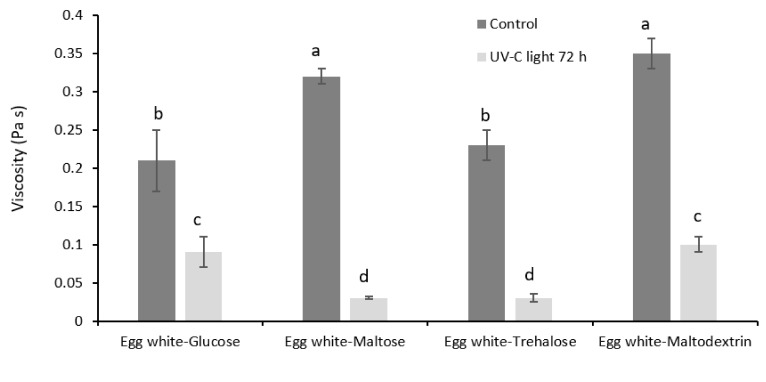
Apparent viscosity of aqueous suspensions of egg white and egg white–sugar mixtures before and after exposure to UV-C light for 72 h. ^a–d^: Means indicated by different letters are significantly different (*p* < 0.05). Error bars represent the Standard Deviation.

**Figure 2:**
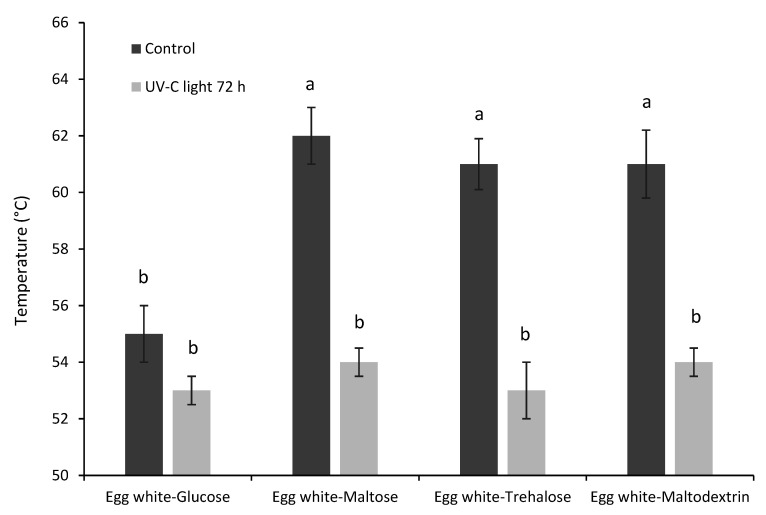
Gelling temperature of aqueous suspensions of egg white–sugar mixtures before and after exposure to UV-C light for 72 h. ^a–d^: Means indicated by different letters are significantly different (*p* < 0.05). Error bars represent the standard deviation.

To understand whether the changes observed upon photoreaction of egg white and carbohydrate could be associated to modification in functional properties, samples were also analyzed for apparent viscosity, gelling and foaming properties. The presence of carbohydrates during exposure to UV-C light promoted a significant decrease in apparent viscosity (Figure 1). This result could be due to the lower hydrodynamic volume of photoinduced glycation particles (Table 3). More compact molecules would beget weaker surface interactions and would be unable to form a relatively stable protein lattice. In this way, the protein network accounting for apparent viscosity would lose the structural continuity. In addition, the novel superficial energy landscape of the glycated products could make them less prone to networking. In other words, it is possible that light exposure would alter the potentiality for surface interaction. This hypothesis is supported by data relevant to gelling temperature (Figure 2), which was found to decrease by circa 8 °C upon UV-C light exposure. The only exception was represented by the sample containing glucose that showed no significant difference despite the different average values of gelling temperature before and after UV-C light exposure. Partially unfolded, cross-linked proteins and glycation products resulted probably in more compact but also more flexible proteins, able to easily form the gel network upon heating [25].

Table 4 shows that exposure to UV-C light also decreased the volume of the foam produced by whipping of the samples prepared from egg white and sugars. This effect could be attributed to the lower apparent viscosity of the suspensions (Figure 1). The lower viscoelasticity of the aqueous phase could be responsible for the formation of a weaker interfacial network during foaming. The only exception was represented by the light-treated sample containing maltodextrin, which produced a higher volume of foam than the untreated one. It is likely that the presence of a large polysaccharide could increase foamability due to its own capacity of surface interaction and jamming in the fluid interstices between bubbles. However, foams prepared from UV-C light treated samples resulted generally more stable than those obtained from the untreated mixtures (Table 4). This result is consistent with previous literature results reporting a lower tendency to bubble dismutation and coalescence in foams obtained from UV-C light treated egg white [10]. It can be hypothesized that photoreacted proteins, having a more compact structure, enclosing carbohydrate moieties and being partially unfolded, could easily denature at the water–air interface, forming a firmer shell around the bubbles and positively affecting their stability.

## 4. Conclusions

Egg white proteins in the presence of sugar and during exposure to UV-C light could undergo non-enzymatic browning reactions. The presence of novel photoinduced protein–carbohydrate components was associated to a decrease in viscosity, a tendency to form gel at lower temperatures and an improved capacity of stabilizing foam interfaces. These data confirm that not only heat treatments but also UV-C light exposure could be exploited to deliver the energy level required to allow interactions and form novel glycation products able to modify the functional properties of proteins. This approach, which does not require the addition of other chemical substances for protein grafting, could thus represent an alternative to conventional wet methods based on heating. In this study we considered as a starting point egg white proteins with well-known peculiar functionalities to demonstrate the applicability of this approach. Nevertheless, other protein sources (e.g., from plants or insects) could be also used to develop novel ingredients with peculiar functionalities that can be exploited in food formulation. Although these results open a new horizon in the possibility to steer functional properties of proteins by using UV-light induced glycation, the applicability of these protein components in food industries will depend on the understanding of their structure–function relationship.

## Figures and Tables

**Table 1:** Color difference (ΔE*) between egg white, sugars and egg white–sugar mixtures exposed to UV-C light for increasing time and the untreated sample. Percentage of ovalbumin reduction is also shown.

	ΔE*			Ovalbumin Reduction (%)		
	4 h	24 h	72 h	4 h	24 h	72 h
Egg white	8.29	17.79	19.41	54.2	75.4	85.5
Glucose	2.15	4.71	5.41	-	-	-
Maltose	4.40	5.27	2.09	-	-	-
Trehalose	7.28	10.43	11.72	-	-	-
Maltodextrin	0.84	1.17	3.76	-	-	-
Egg white–Glucose	15.36	23.18	24.35	28.5	53.6	65.6
Egg white–Maltose	10.91	20.97	24.86	46.2	50.1	64.3
Egg white–Trehalose	12.00	20.32	24.74	47.6	63.2	72.1
Egg white–Maltodextrin	11.30	19.71	22.07	67.1	72.6	75.3

For ΔE* values: DS < 0.05. For % of ovalbumina reduction: DS < 2%.

**Table 2:** Mass percentage (±standard deviation) flowing downstream upon filtration of freeze-dried egg white, sugars and egg white–sugar mixtures before and after exposure to UV-C light for 72 h (UV-C light).

	Mass (%)	
	Control	UV-C Light 72 h
Egg white	70 ± 2	17 ± 1
Egg white–Glucose	80 ± 1	63 ± 1
Egg white–Maltose	91 ± 1	52 ± 2
Egg white–Trehalose	95 ± 5	52 ± 2
Egg white–Maltodextrin	81 ± 1	43 ± 3

**Table 3:** Critical concentration and hydrodynamic radius (±population dispersion around the peak) of particles relevant to populations I and II in aqueous suspensions of egg white and egg white–sugar mixtures before and after exposure to UV-C light for 72 h. Population percentage in brackets.

Sample		Egg White	Egg White–Glucose	Egg White–Threalose	Egg White–Maltose	Egg White–Maltodextrin
Control	Critical Concentration (g/L)	14	1667	667	130	67
	Population I (nm)	193.6 ± 134.3 (16.1)	62.4 ± 23.9 (17.9)	71.4 ± 37.3 (21.3)	63.9 ± 71.0 (36.8)	94.7 ± 57.6 (15.7)
	Population II (nm)	4733.9 ± 3305.5 (83.9)	4347.3 ± 1310.2 (82.1)	5517.7 ± 1832.4 (78.7)	5198.0 ± 3958.4 (63.2)	8161.7 ± 13.0 (84.3)
UV-C light 72 h	Critical Concentration (g/L)	1200	2023	2488	547	670
	Population I (nm)	202.4 ± 220.8 (33.1)	170.2 ± 27.7 (86.2)	108.7 ± 66.5 (31.9)	167.3 ± 21.4 (45.1)	11.5 ± 10.1 (7.5)
	Population II (nm)	7265.0 ± 2745.1 (69.9)	2738.9 ± 577.9 (13.8)	4941.8 ± 3706.3 (68.1)	2968.6 ± 1161.4 (55.0)	360.8 ± 319.3 (92.5)

**Table 4:** Foam ability and stability (±standard deviation) of aqueous suspensions of egg white–sugar mixtures before and after exposure to UV-C light for 72 h.

	Foam Ability (%)		Foam Stability (%)	
	Control	UV-C Light 72 h	Control	UV-C Light 72 h
Egg white–Glucose	41.0 ± 0.0	30.6 ± 1.6	77.8 ± 0.0	86.8 ± 1.6
Egg white–Maltose	40.7 ± 0.4	35.9 ± 0.7	78.2 ± 0.6	88.6 ± 1.6
Egg white–Trehalose	45.8 ± 0.6	35.9 ± 0.7	89.1 ± 1.2	88.6. ± 1.6
Egg white–Maltodextrin	35.8 ± 0.6	50.2 ± 0.3	75.9 ± 1.3	91.1 ± 0.3

## Data Availability

The data presented in this study are available on request from the corresponding author.
